# Angiogenesis Inhibitors for Head and Neck Squamous Cell Carcinoma Treatment: Is There Still Hope?

**DOI:** 10.3389/fonc.2021.683570

**Published:** 2021-06-14

**Authors:** Aini Hyytiäinen, Wafa Wahbi, Otto Väyrynen, Kauko Saarilahti, Peeter Karihtala, Tuula Salo, Ahmed Al-Samadi

**Affiliations:** ^1^ Department of Oral and Maxillofacial Diseases, Clinicum, University of Helsinki, Helsinki, Finland; ^2^ Translational Immunology Programme, Faculty of Medicine, University of Helsinki, Helsinki, Finland; ^3^ Department of Oncology, Helsinki University Hospital and University of Helsinki, Helsinki, Finland; ^4^ Department of Oncology, Helsinki University Hospital Comprehensive Cancer Centre and University of Helsinki, Helsinki, Finland; ^5^ Department of Pathology, University of Helsinki, Helsinki, Finland; ^6^ Cancer Research and Translational Medicine Research Unit, University of Oulu, Oulu, Finland; ^7^ Oulu Medical Research Centre, Oulu University Hospital, University of Oulu, Oulu, Finland

**Keywords:** anti-angiogenesis, head and neck cancer, therapy, endostatin, bevacizumab

## Abstract

**Background:**

Head and neck squamous cell carcinoma (HNSCC) carries poor survival outcomes despite recent progress in cancer treatment in general. Angiogenesis is crucial for tumour survival and progression. Therefore, several agents targeting the pathways that mediate angiogenesis have been developed. We conducted a systematic review to summarise the current clinical trial data examining angiogenesis inhibitors in HNSCC.

**Methods:**

We carried out a literature search on three angiogenesis inhibitor categories—bevacizumab, tyrosine kinase inhibitors and endostatin—from Ovid MEDLINE, Cochrane Library, Scopus and ClinicalTrials.gov database.

**Results:**

Here, we analysed 38 clinical trials, total of 1670 patients, investigating 12 angiogenesis inhibitors. All trials were in phase I or II, except one study in phase III on bevacizumab. Angiogenesis inhibitors were used as mono- and combination therapies together with radio-, chemo-, targeted- or immunotherapy. Among 12 angiogenesis inhibitors, bevacizumab was the most studied drug, included in 13 trials. Although bevacizumab appeared effective in various combinations, it associated with high toxicity levels. Endostatin and lenvatinib were well-tolerated and their anticancer effects appeared promising.

**Conclusions:**

Most studies did not show benefit of angiogenesis inhibitors in HNSCC treatment. Additionally, angiogenesis inhibitors were associated with considerable toxicity. However, some results appear encouraging, suggesting that further investigations of angiogenesis inhibitors, particularly in combination therapies, for HNSCC patients are warranted.

**Systematic Review Registration:**

PROSPERO (https://www.crd.york.ac.uk/prospero/), identifier CRD42020157144.

## Introduction

Head and neck squamous cell carcinoma (HNSCC) is the eighth most common neoplasm worldwide with more than 600 000 new cases and 350 000 deaths reported in 2018 (1). HNSCC can arise from subsites within the oral cavity, oropharynx, hypopharynx, larynx and nasopharynx (2). The most common risk factors include excess tobacco and alcohol consumption and human papillomavirus (HPV) infection (2). Currently, the primary treatment of HNSCC patients consists of surgery and (chemo-) radiotherapy either alone or in combination (3). Despite intensive research and progress in cancer therapy, survival outcomes in patients with locoregionally advanced disease remains poor, with a five-year overall survival (OS) rate reaching only around 50% (4).

Angiogenesis (neo-angiogenesis, new blood vessel formation) is crucial for tumour growth, invasion and metastasis (5). Angiogenesis is a hallmark of tumour progression and has been studied in many cancer types, including HNSCC (6). Angiogenesis is primarily mediated by the vascular endothelial growth factor (VEGF) pathway (7). Two major categories of agents have been developed to target this pathway: antibody-based agents and VEGF receptor tyrosine kinase inhibitors (TKIs) (6). The US Food and Drug Administration (US FDA) has approved several anti-angiogenic agents to treat solid tumours, such as colorectal cancer, renal cell carcinoma, ovarian cancer, gastric cancer and thyroid cancer (7). Contrary to concerns that angiogenesis inhibitors could increase hypoxia and lead to treatment resistance, these inhibitors in preclinical models appear to overcome resistance and preclinically synergise with traditional therapies such as radiation (8). Paradoxically, such therapies normalise tumour vasculature, increase tumour blood flow and reduce hypoxia, and, thus, carry synergistic effects with radiation and chemotherapy (8, 9).

Despite the crucial role of angiogenesis in HNSCC, as yet no anti-angiogenic agent enjoys clinical use for these patients, and conclusive data from clinical trials on anti-angiogenic drugs in HNSCC remain unavailable. This systematic review aims to summarise the current data from clinical trials on three angiogenesis inhibitor categories (bevacizumab, TKIs and endostatin) in HNSCC patients.

## Materials and Methods

### Protocol and Registration

This review was registered at the international prospective register of systematic reviews PROSPERO (https://www.crd.york.ac.uk/prospero/) under registration number CRD42020157144.

### Search Strategy

Since there is no clear definition of angiogenesis inhibitors and several compounds with anti-angiogenic effects together with other antitumour effects exist, here we included only the three primary classifications of angiogenesis inhibitors: bevacizumab, TKIs and endostatin.

We conducted a literature search in November 2019 using three databases (Ovid MEDLINE, Cochrane Library and Scopus) and the National Library of Medicine website (https://ClinicalTrials.gov). We conducted a literature search in November 2019 using three databases (Ovid MEDLINE, Cochrane Library and Scopus) and the National Library of Medicine website (https://ClinicalTrials.gov). We used the following search terms: (“head and neck cancer” OR “head and neck squamous cell carcinoma”) AND (“angiogenesis inhibitors” OR bevacizumab OR avastin OR “Bayer 205” OR semaxanib OR su5416 OR thrombospondin OR abt-510 OR pazopanib OR votrient OR sunitinib OR su11248 OR su11248 OR sorafenib OR nexavar OR ranibizumab OR lucenti OR endostatin OR ramucirumab OR cyramza OR vandetanib OR zd6474 OR zactima OR axitinib OR inlyta OR cabozantinib OR cometriq OR cabometyx OR lenvatinib OR lenvima OR regorafenib OR ziv-aflibercept OR zaltrap OR “VEGFR antagonists” OR VEGF OR “vascular endothelial growth factor”) AND (“randomized controlled trials” OR “clinical trials”). We gathered the search results in Mendeley, and used the Preferred Reporting Items for Systematic Reviews and Meta-Analyses (PRISMA) to illustrate the results in a flowchart (10). We excluded any duplicates and articles that did not meet the listed inclusion criteria (**Supplementary Table 1**). Three independent researchers (AH, WW and OV) carried out the literature search, screened all retrieved article titles and abstracts, discarded duplicates and verified that the included articles satisfied our inclusion criteria. Three articles were published after the search was completed (11–13) and were subsequently added to our review.

### Data Extraction

For the included articles, we extracted the following information: (1) basic article information including first author, publication year, trial year, trial phase, treatment setting and follow-up time period; (2) patient and tumour characteristics including the number of patients treated and the cancer type; (3) trial methods including regimens for each treatment arm and evaluation criteria; (4) treatment effect and survival information including complete response rate (CR), partial response rate (PR), overall response rate (ORR), stable disease (SD), progressive disease (PD), disease control rate (DCR), overall survival (OS) and progression-free survival (PFS); (5) toxicity of the treatment; and (6) study conclusions.

## Results

### Search Results

**Figure 1:**
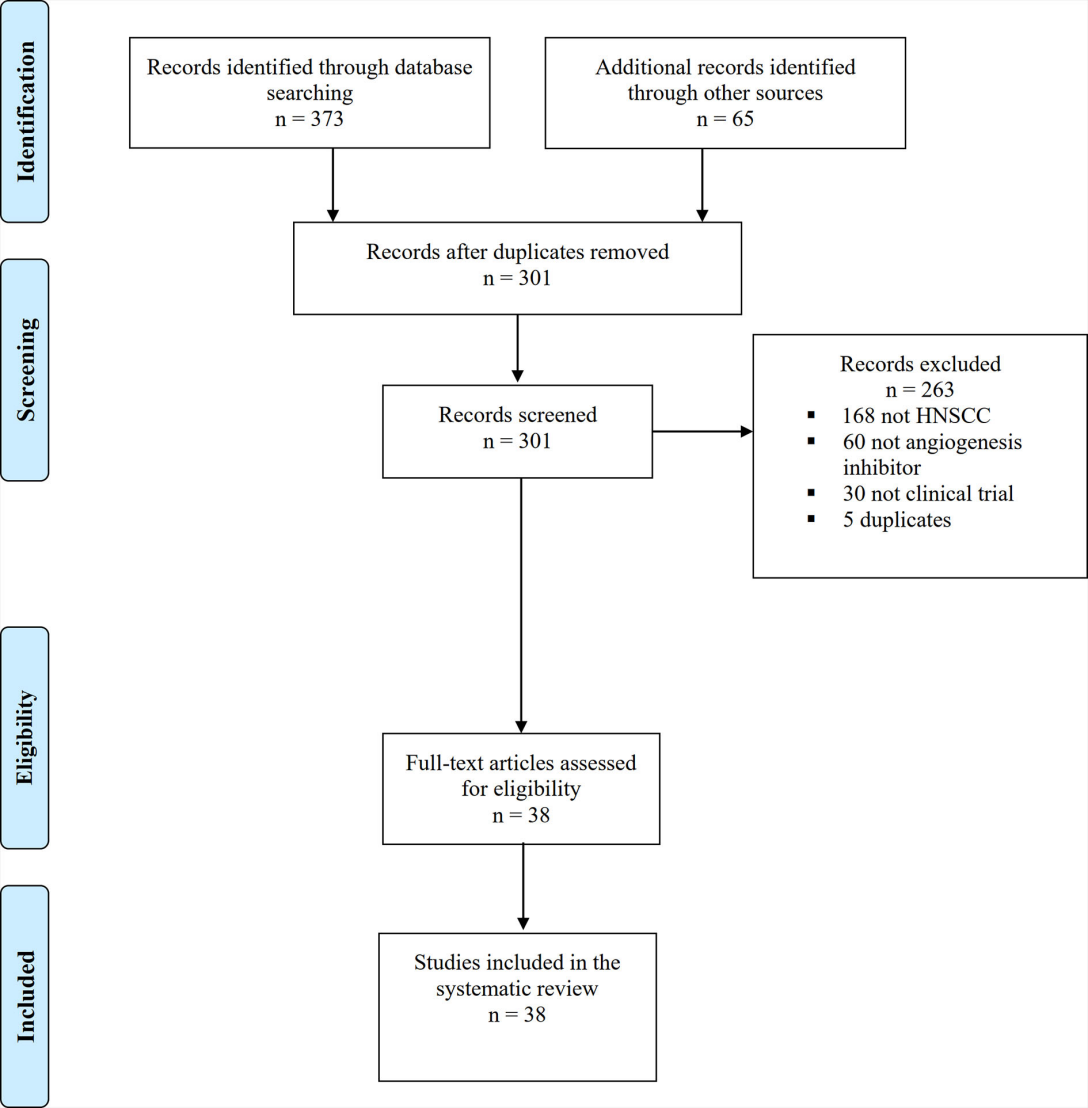
PRISMA flow chart with search results and studies included and excluded in different steps.

We found a total of 373 articles from our database search (234 from Ovid MEDLINE, 84 from Cochrane Library and 55 from Scopus), 62 from our ClinicalTrials.gov search and three articles were published after the initial search and subsequently added to the systematic review (**Figure 1**). From these, 38 articles met the inclusion criteria and were included in this systematic review. All clinical trials were carried out on patients with recurrent, metastatic or locally advanced HNSCC.

### Bevacizumab

Bevacizumab, the first US FDA-approved angiogenesis inhibitor, is a humanised monoclonal antibody against VEGF-A. Approved by the US-FDA as a first-line treatment for metastatic colorectal cancer and for other malignancies such as advanced non-squamous non-small cell lung cancer (NSCLC), ovarian cancer, renal cell carcinoma (RCC) and glioblastoma multiforme, it is used alone or in combination with other treatments (14, 15). Bevacizumab was the most frequently studied angiogenesis inhibitor in HNSCC featured in a total of 13 trials among 866 patients (**Table 1**). The largest trial was a phase III (11), while the remainder were phase I or II trials. Eleven trials used bevacizumab as a first-line treatment, one trial used it as a first- or second-line treatment and one trial used it as second-line treatment. Bevacizumab was used in combination with different therapies administered in doses of 10–15 mg/kg.

**Table 1:** Summary of the bevacizumab clinical trials for head and neck squamous cell carcinoma.

Reference, clinical trial number	Intervention	Phase	Completion year, Country	Treatment line	No. of patients	Follow-up	Evaluation criteria	CR	PR	ORR	SD	PD	DCR	OS	PFS	Conclusion
11	**Control group:** Platinum-containing chemotherapy	III	2015, USA	First	**Total:** n = 403	Median 40 months	RECIST	N/A	N/A	**Control group:** 24.5%	N/A	N/A	N/A	**Median OS (months)**	**Median PFS (months)**	“The addition of bevacizumab to chemotherapy did not improve OS but improved the response rate and progression-free survival with increased toxicities. These results encourage biomarker-driven studies of angiogenesis inhibitors with better toxicity profiles in select patients with SCCHN.”
					**Control group:** n = 200									11.0 in control and 12.6 in bevaci-zumab group (HR 0.87 95% CI 0.70-1.09 p-value 0.22)	4.3 in control and 6.0 in bevazicu-mab group (p-value 0.0014)	
NCT00588770	**Bevacizumab group:**									**Bevaci-zumab group:**						
	Platinum-containing chemo-therapy with bevacizumab				**Bevaci-zumab group:** n = 203					35.5% (p-value 0.016)				**Control group:**	**Control group:**	
														2-year OS	2-year PFS	
														25.2%	2.1%	
														3-year OS	3-year PFS	
														16.4%	0.5%	
														4-year OS	4-year PFS	
														11.8%	0.5%	
	Platinum chemo therapy regimens:													**Bevacizumab group:**	**Bevaci-zumab group:**	
	(1) docetaxel + cisplatin, (2) docetaxel + carboplatin, (3) cisplatin + FU or (4) carbo-platin + FU													2-year OS	2-year PFS	
														18.1%	7.1%	
														3-year OS	3-year PFS	
														10.0%	5.5%	
														4-year OS	4-year PFS	
														6.4%	3.7%	
16	Bevacizumab with cisplatin, docetaxel, 5-fluorourail, erlotinib and radiotherapy	I	N/A, USA	First	n = 13	Median 23.4 months	RECIST	N/A	N/A	N/A	N/A	N/A	N/A	2-year OS 54%	N/A	“Erlotinib in combination with induction TPF followed by erlotinib, cisplatin and bevacizumab with XRT is active but toxic. Gastrointestinal toxicities partly caused high rates of study withdrawal. All doses studied in this protocol caused unexpected toxicities and we do not recommend advancement to phase II.”
17		II	N/A, USA	First	**Total:** n = 78	Median 32 months	RECIST 1.1	N/A	N/A	N/A	N/A	N/A	N/A	2 year OS in combinedcohorts 0.88 (95% CI 0.81-0.96)	2 year PFS	“RT with a concurrent non-platinum regimen of cetuximab and pemetrexed is feasible in academic and community settings, demonstrating expected toxicities and promising efficacy. Adding bevacizumab increased toxicity without apparent improvement in efficacy, countering the hypothesis that dual EGFR–VEGF targeting would overcome radiation resistance, and enhance clinical benefit. Further development of cetuximab, pemetrexed, and RT will require additional prospective study in defined, high-risk populations where treatment intensification is justified.”
NCT0070397	**Control group:**				**Control group:**										**Control group:**	
	Cetuximab, pematrexed and radiotherapy				n = 37										79% (95% CI 0.69-0.92 p-value <0.0001)	
	**Bevacizumab group:** Cetuximab, pematrexed and radiotherapy with bevacizumab				**Bevaciz-umab group:** n = 41										**Bevaci-zumab group:** 75% (95% CI 0.64-0.88 p-value <0.0001)	
18	Bevacizumab with cisplatin, cetuximab and radio therapy	II	2013, USA	First	n = 30	Median 33.8 months	N/A	N/A	N/A	N/A	N/A	N/A	N/A	2-year OS: 92.8% (95% CI 74.2-98.1)	2-year PFS: 88.5% (95% CI 68.1-96.1)	“The addition of bevacizumab and cetuximab to two cycles of cisplatin, given concurrently with IMRT, was well tolerated and was associated with favorable efficacy outcomes in this patient population.”
19	Bevacizumab with cisplatin and radiotherapy	I	2010, USA	First	n = 10	Mean 61.3 months	PER-CIST	N/A	N/A	N/A	N/A	N/A	N/A	At the last follow-up visit, 9 of 10 patients were alive.	Median PFS 50.1 months	“The incorporation of bevacizumab into comprehensive chemoradiation therapy regimens for patients with HNSCC appears safe and feasible. Experimental imaging demonstrates measureable changes in tumor proliferation, hypoxia, and perfusion after bevacizumab monotherapy and during chemoradiation therapy. These findings suggest opportunities to preview the clinical outcomes for individual patients and thereby design personalized therapy approaches in future trials.”
20	Bevacizumab with cetuximab	II	2008, USA	First	n = 30	Median 38 months	N/A	N/A	N/A	N/A	N/A	N/A	3 year OS	3 year PFS	Median	“The combination of bevacizumab, docetaxel, and RT is tolerable and effective in HNSCC. This regimen is worthy of further study in appropriate subset of patients receiving chemoradiation therapy.”
NCT00281840													68.2% (95% CI: 47.5–82.1%)	61.7% (95% CI: 41.5–75.7%)	2.8 months (95% CI 2.7-4.2 months)	
21	Bevacizumab with cetuximab	II	2010, USA	First or second	**Total:** n = 46	Median 9.7 months	RECIST	N/A	16% (n = 7)	16% (95% CI 7-24%)	58% (n = 26)	N/A	73% (n = 33)	Median	Median	“Cetuximab and bevacizumab are supported by preclinical observations and are well tolerated and active in previously treated patients with SCCHN.”
					**Evaluated patients:** n = 45									7,5 months (95% CI 5.7-9.6 months)	2,8 months (95% CI 2.7-4.2 months)	
22	Bevacizumab with cisplatin and radiotherapy	II	N/A, USA	First	n = 44	Median 2.5 years	N/A	N/A	N/A	N/A	N/A	N/A	N/A	2-year OS	2-year PFS	“It was feasible to add bevacizumab to chemoradiation for NPC treatment. The favorable 2-year OS of 90.9% suggests that bevacizumab might delay progression of subclinical disease.”
														90.9% (95% CI 82.3-99.4)	74.7% (95% CI 91.8-87.6)	
23	Bevacizumab and erlotinib with concurrent cisplatin and radio-therapy	I	2010, USA	First	n = 28	Median 46 months	N/A	96% (95% CI 82- 100%)	N/A	N/A	N/A	N/A	N/A	3-yearOS	3-year PFS	“The current study shows acceptable safety and encouraging efficacy with the integration of dual EGFR and VEGF inhibitors with CRT in locally advanced nonmetastatic HNC. The increased incidence of osteoradionecrosis and soft tissue necrosis may be associated with the use of bevacizumab. These results warrant further study in a larger multi-institutional and/or randomized setting.”
NCT00140556														86% (95% CI 66-94%)	82% (95% CI 62-92%)	
24	Bevacizumab with cisplatin and radiotherapy	II	2011, USA	First	n = 42	Median 31.8 months	N/A	N/A	N/A	N/A	N/A	N/A	N/A	2-year OS	2-year PFS	“The addition of bevacizumab and cetuximab to two cycles of cisplatin, given concurrently with IMRT, was well tolerated and was associated with favorable efficacy outcomes in this patient population.”
														88% (95% CI 78.6%-98.4%)	75,9% (95% CI 63.9%-90.1)	
25	Bevacizumab, paclitaxel, radiotherapy and erlotinib Neoadjuvant therapy (6 weeks): paclitaxel, carboplatin, 5-fluorouracil and bevacizumab	II	2008, USA	First	**Total:** n = 60	Median 32 months	RECIST	30% (n = 16)	65% (n = 35)	65% (n = 35)	35% (n = 19)	N/A	N/A	2-yearOS	2-year PFS	“The addition of bevacizumab and erlotinib to first-line combined modality therapy was feasible in a community- based setting, producing toxicity comparable to other effective combined modality regimens for head and neck cancer. The high level of efficacy suggests that incorporation of these targeted agents into first-line therapy should be further explored.”
					**Evaluate d patients:** n = 54				(95% CI 52-78%)	(95% CI 52%-78%)				90%	83%	
														3-year OS	3-year PFS	
														82%	71%	
26	**Control group (FHX):** 5-fluorouracil, hydroxyurea and radiotherapy	II	2007, USA	First	**Total:** n = 26	Median 29 months	RECIST		N/A	N/A	N/A	N/A	N/A	2-year OS	N/A	“Locoregional progression seen in T4N0-1 tumors treated with BFHX was unexpected and led to study termination. The addition of bevacuzimab to chemoradiotherapy for HNSCC should be limited clinical trials.”
	**Bevacizumab group (BFHX):** Bevacizumab, 5-fluoroura-cil, hydroxy- urea and radiotherapy				**FHX group:** n = 9			**FHX group:** 100% (n = 8)						**FHX group:** 89% (95% CI 43-98)		
					**BFHX group:** n = 17			**BFHX group:** 86% (n = 12)						**BFHX group:** 58% (95% CI 33-78)		
					**Evaluated patients:** n = 22											
					**FHX group (evaluated)** n = 8											
					**BFHX group: (evaluated)** n = 14											
27	Bevacizumab and erlotinib in escalating dose cohorts	I, II	2005, USA	Second	**Total:** n = 56	N/A	RECIST	15% (n = 7)	N/A	15%	31% (n= 15)	N/A	N/A	Median 7.1 months (95% CI 5.7-9.0)	Median 4.1 months (95% CI 2.8-4.4)	“The combination of erlotinib and bevacizumab is well tolerated in recurrent or metastatic squamous cell carcinoma of the head and neck. Some patients appear to derive a sustained benefit and complete responses were associated with expression of putative targets in prettreatment tumor tissue.”
					**Phase 1:** n = 10			(95% CI 6-28%)								
					**Phase 2:** n = 46											

BFHX, 5-fluorouracil, hydroxyurea, radiation and bevacizumab; CDR, disease control rate; CI, confidence interval; CR, complete response rate; CRT, chemoradiation; EGFR, epidermal growth factor receptor; HNC, head and neck cancer; HNSCC, head and neck squamous cell carcinoma; HR, hazard ratio; IMRT, intensity-modulated radiation therapy; N/A, not available; NPC, nasopharyngeal carcinoma; ORR, overall response rate; OS, overall survival; PD, progressive disease; PFS, progression free survival; PR, partial response rate; RECIST, Response Evaluation Criteria in Solid Tumours; RT, radiotherapy; SCCHN, squamous cell carcinoma of the head and neck; SD, stable disease; VEGF, vascular endothelial growth factor; WHO, World Health Organization criteria; XRT, radiotherapy.

In the phase III study, 403 patients were randomly assigned to receive platinum-based chemotherapy with or without bevacizumab as a first-line treatment (11). There was a minor but statistically nonsignificant increase in median OS (median OS with chemotherapy 11.0 months and with the addition of bevacizumab 12.6 months; hazard ratio (HR) 0.87; 95% CI 0.70–1.09; p=0.22). The addition of bevacizumab to chemotherapy improved PFS and response rates significantly. Median PFS was 6.0 months with bevacizumab + chemotherapy and 4.3 months with chemotherapy alone (p=0.0014). ORR was 35.5% in the bevacizumab + chemotherapy group and 24.5% in the chemotherapy only group (p=0.016). The addition of bevacizumab increased toxicities.

Four trials, three as first-line treatment, combined bevacizumab and epidermal growth factor TKI erlotinib with different types of chemotherapy or chemoradiotherapy. The combination of bevacizumab, erlotinib and chemoradiotherapy (cisplatin, docetaxel and 5-fluorouracil) was active, but toxic (16). In this phase I study, gastrointestinal toxicities caused high rates of patient withdrawal and the combination was not recommended to advance to phase II (16). The other studies with bevacizumab and erlotinib, however, showed more promising results. Bevacizumab and erlotinib with concurrent cisplatin and radiotherapy demonstrated an encouraging efficacy with acceptable safety in nonmetastatic, locally advanced HNSCC (23). Complete response rates were achieved in 96% of patients (95% CI 82–100%) and 3-year OS and PFS reached 86% and 82%, respectively (95% CI 66–94% and 62–92%) (23). Neoadjuvant therapy (6 weeks) consisting of paclitaxel, carboplatin, 5-fluorouracil and bevacizumab followed by bevacizumab and erlotinib in combination with radiotherapy and paclitaxel showed good efficacy and proved safe (25). ORR was 65% (95% CI 52–78%) and 2-year OS and PFS were 90% and 83%, respectively (95% CI 78.6–98.4% and 63.9–90.1%). Bevacizumab and erlotinib were also studied in escalating dose cohorts as the second-line treatment for metastatic or recurrent HNSCC (27). The combination was well-tolerated with a median OS of 7.1 months (95% CI 5.7–9.0 months) and PFS of 4.1 months (95% CI 2.8–4.4 months) (27).

In addition, bevacizumab was combined with cetuximab in three trials. The addition of bevacizumab to cetuximab, pemetrexed and radiotherapy as a first-line treatment increased toxicities without apparent improvement in efficacy (17). Moreover, 2-year PFS was 79% with cetuximab + chemoradiotherapy and 75% when bevacizumab was added (95% CI 0.69–0.92%for the control group and 0.64–0.88% for the bevacizumab group) (17). Bevacizumab and cetuximab with cisplatin and radiotherapy as a first-line treatment was well-tolerated with favourable survival rates, where 2-year OS and PFS reached 92.8% and 88.5%, respectively (95% CI 74.21–98.1% and 68.1–96.1%) (18). In a clinical trial examining bevacizumab and cetuximab in metastatic or recurrent HNSCC as the first- or second-line treatment, an ORR of 16% was reported (95% CI 7–24%) with a median OS and PFS of 7.5 and 2.8 months, respectively (95% CI 5.7–9.6 and 2.7–4.2 months). The treatment was well-tolerated (21).

In five clinical trials, bevacizumab was combined with chemoradiation as a first-line treatment. Bevacizumab with 5-fluorouracil, hydroxyurea and radiotherapy proved toxic and the study was interrupted early after adding bevacizumab to chemoradiation led to acute toxicity (26). The CR rate with chemoradiation alone was 100% compared to 86% when chemoradiation was combined with bevacizumab. In addition, 2-year OS reached 89% (95% CI 43–98%) in the control group falling to 58% (95% CI 33–78%) in the bevacizumab group (26). Other trials reported more promising results. For instance, bevacizumab combined with docetaxel and radiotherapy was well-tolerated and effective with a 3-year OS of 68.2% (95% CI 47.5–82.1%) and a PFS of 61.7% (95% CI 41.5–75.7%) (20). Adding bevacizumab to cisplatin and radiotherapy was also well-tolerated and studied in three trials. In a phase II trial, 2-year OS was 88% (95% CI 78.6–98.4%), while 2-year PFS was 75.9% (95% CI 63.9–90.1%) (24). Another phase II trial also reported encouraging survival rates of a similar magnitude: 2-year OS was 90.9% (95% CI 82.3–99.4%) and 2-year PFS was 74.7% (95% CI 91.8–87.6%) (22). Finally, a phase I trial reported a median PFS of 50.1 months (19).

To summarise, three studies with bevacizumab reported significant toxicities with no treatment efficacy: one in combination with erlotinib and chemoradiotherapy, one with cetuximab and chemoradiotherapy and one with chemoradiotherapy. The other ten trials reported more acceptable safety profiles and efficacy. **Supplementary Table 2** summarises the toxicity analysis of bevacizumab.

### Sorafenib

Sorafenib (BAY-43-9006) is an anticancer drug approved by the US FDA to treat unresectable hepatocellular carcinoma, radioactive iodine refractory thyroid cancer and advanced renal cell carcinoma (28). The anticancer effects of sorafenib are mediated primarily by targeting both the RAF/MEK/ERK pathway and the receptor tyrosine kinases, including VEGFR (VEGFR-1, VEGFR-2 and VEGFR-3), platelet-derived growth factor receptor (PDGFR), FLT3, Ret and c-KIT (29, 30).

Sorafenib was studied in 5 phase II clinical trials among a total of 201 patients (**Table 2**) three times as a monotherapy and twice in combination with cisplatin, 5-fluorouracil and cetuximab. The sorafenib dose across all trials was 400-mg twice daily as a continuous treatment. As a single agent in the first- or second-line advanced setting, ORR of 3.7% was reported (95% CI 0.1–19%) with a median OS of 4.2 months (95% CI 3.6–8.7 months) (35). In the other two monotherapy studies, ORR was not reported, although the median OS was 8.0 months when administered to patients who primarily received previous chemo- and/or radiotherapy and 9.0 months when administered as a first-line treatment (95% CI 2.4–9.8 and 7–14 months), with a median PFS of 3.4 and 4.0 months, respectively (95% CI 1.8–4 and 2–4 months) (32, 34). Sorafenib in combination with cetuximab demonstrated no clinical benefit with an ORR of 8% and median OS or PFS of 5.7 and 3.2 months, respectively (31). The combination of sorafenib with cisplatin and 5-fluorouracil emerged as a feasible regimen as a first-line treatment with an ORR of 77.8% and median OS and PFS of 11.8 and 7.2 months, respectively (33). Overall, sorafenib was well-tolerated with a modest anticancer activity. **Supplementary Table 2** summarises the toxicity analysis for sorafenib.

**Table 2:** Summary of the sorafenib clinical trials for head and neck squamous cell carcinoma.

Reference, clinical trial number	Intervention	Phase	Completion year, Country	Treatment line	No. of patients	Follow-up	Evaluation criteria	CR	PR	ORR	SD	PD	DCR	OS	PFS	Conclusion
31	**Control group:** Cetuximab	II	2011, USA	First or more	**Total:** n = 55	N/A	RECIST	N/A	N/A	**Control group:** 8%	N/A	N/A	N/A			“In summary, our study demonstrated that sorafenib did not add clinical benefit to cetuximab alone but added toxicities, and was terminated early base on a planned interim analysis. Our correlative studies suggest that patients with p16-negative tumors or low plasma TGFβ1 expression may derive benefits from cetuximab-based therapy. In addition, patients with high plasma TGFβ1 expression may potentially benefit from TGFβ pathway targeted agents or immune checkpoint inhibitors in combination with cetuximab. However, these are very exploratory findings, and further studies are warranted.”
					**Control group:** n = 27									**Control group:** Median 9 months (95% CI 5.2-12.9)	**Control group:** Median 3 months (95% CI 1.9-5.0)	
	**Sorafenib group:** Cetuximab with sorafenib				**Sorefanib group:** n = 28					**Sorefanib group:** 8%				**Sorefanib group:** Median 5.7 months (95% CI 4.2-10.8)	**Sorafenib group:** Median 3.2 months (95% CI 1.8-4.2)	
(32)	Sorafenib monotherapy	II	N/A, Belgium	Second or more	**Total:** n = 24	N/A	RECIST	0%	5% (n = 1)	N/A	55% (n = 12)	40% (n = 1.8-4 9)	N/A	Median 8.0 months (95%CI 2.4–9.8)	Median 3.4 months (95% CI 1.8–4)	“In conclusion, data from this phase II trial suggest that sorafenib provides only a modest cytostatic efficacy in patients with recurrent SCCHN. Only a minority of patients showed a prolonged disease control of more than 4 months. Therefore, further studies with this single agent in unselected patient’s population are not warranted.”
NCT00199160					**Evaluated patients:** n = 22											
33	Sorafenib with cisplatin and 5-fluoro-uracil	II	2011, China	First	n = 54	Median 19.0 months	RECIST	1.9% (n = 1)	75.9% (n = 41)	77.80% (n = 42)	13.0% (n = 7)	9.2% (n = 5)	90.80% (n = 49)	Median 11.8 months (95% CI 10.6-18.7)	Median 7.2 months (95% CI 6.8-8.4)	“Combination of sorafenib, cisplatin and 5-FU was tolerable and feasible in recurrent or metastatic NPC. Further randomized trials to compare sorafenib plus cisplatin and 5-FU with standard dose of cisplatin plus 5-FU in NPC are warranted.”
34	Sorafenib monotherapy	II	2006, USA	First	n = 41	N/A	RECIST	N/A	n = 1	N/A	N/A	N/A	51%	Median 9 months (95% CI 7-14)	Median 4 months (95% CI 2-4)	“Although response was poor, progression-free and overall survival times compare favorably with previous Southwest Oncology Group, phase II, single-agent trials.”
									2% (95% CI 0-13%)				(95% CI 35-67%)			
35	Sorafenib monotherapy	II	N/A, Canada	First or second	**Total:** n = 27	N/A	RECIST	N/A	3.7% (n = 1)	3.7% (n = 1)	37.0% (n = 10)	37.0% (n = 10)	40.7%	Median 4.2 months	6-month PFS	“Sorafenib was well tolerated and had modest anticancer activity comparable to monotherapy with other targeted agents in this group of patients. Further development in combination with radiation or other agents may be warranted.”
					**Evaluate d patients:** n = 26					(95% CI 0.1-19)	(95% CI 19.4-57.6)			(95% CI 3.6-8.7)	3.9% (95% CI 0.6-26.4)	

CDR, disease control rate; CI, confidence interval; CR, Complete response rate; HR, hazard ratio; N/A, not available; NPC, nasopharyngeal carcinoma; ORR, overall response rate; OS, overall survival; PD, progressive disease; PFS, progression free survival; PR. Partial response rate; RECIST, Response Evaluation Criteria in Solid Tumours; SCCHN, squamous cell carcinoma of the head and neck; SD, stable disease; TGFβ1, Transforming growth factor beta 1; WHO, World Health Organization criteria.

### Sunitinib and Semaxinib

Sunitinib (SU11248) inhibits multiple receptor tyrosine kinases including VEGFR-1, -2 and -3, KIT, foetal liver tyrosine kinase receptor 3 (FLT3), PDGFRα and PDGFRβ, colony-stimulating factor receptor type 1 (CSF1R) and the glial cell line–derived neutrophilic factor receptor (GDNF) (33, 36). Approved by the US FDA, sunitinib treats advanced renal cell carcinoma and advanced gastrointestinal stromal tumours (37). Semaxinib (SU5416), a predecessor of sunitinib, has poor pharmacological properties and limited efficacy (38).

Sunitinib was studied in 4 trials among a total of 91 patients (**Table 3**): in two as a monotherapy, in one after platinum-based chemotherapy and in one in combination with bortezomib (**Table 3**). No ORR was reported in any of these studies. In the only trial that treated patients using sunitinib as a first-line treatment, no objective responses were observed and the trial was discontinued prematurely (42). As a monotherapy, 1-year OS rates of 22% and 14% (22% in patients with Eastern Cooperative Oncology Group Performance Status (PS) 0–1 and 14% with PS 2) were reported, while in another monotherapy study, median OS and PFS reached 102 and 60 days, respectively (40, 41). After prior platinum-based chemotherapy, a clinical benefit rate (CBR = SD + PR + CR) was achieved in 28.6% of patients, with no CR, while median OS and PFS reached 10.5 and 3.5 months, respectively (39). Treatment with sunitinib was well-tolerated, although the anticancer effect remained modest.

**Table 3:** Summary of the sunitinib and semaxanib clinical trials for head and neck squamous cell carcinoma.

Reference, clinical trial number	Intervention	Phase	Completion year, Country	Treatment line	No. of patients	Follow-up	Evaluation criteria	CR	PR	ORR	SD	PD	DCR	OS	PFS	Conclusion
39	Sunitinib after prior platinum-based chemotherapy	II	2008, China	Second	n = 14	Median 23.1 months	RECIST	0	7,1%	N/A	21%	N/A	N/A	Median	Median	“Sunitinib demonstrated modest clinical activity in heavily pretreated NPC patients. However, the high incidence of hemorrhage from the upper aerodigestive tract in NPC patients who received prior high-dose RT to theregion is of concern. Direct vascular invasion by tumors appeared to increase the risk of serious bleeding.”
														10.5 months (95% CI 7.2-20.7)	3.5 months (95% CI 2.5-9.4)	
									n = 1		(n = 3)			1-year OS		
														35.7%		
40	Sunitinib monotherapy Patients where divided into two cohorts based on their ECOG performance status (PS):	II	N/A, USA	Second	**Total** n = 22	N/A	RECIST	N/A		N/A		N/A	N/A	Median OS	N/A	“Sunitinib had low single agent activity in SCCHN necessitating early closure of cohort A at interim analysis. Sunitinib was well tolerated in PS 2 patients. Further evaluation of single agent sunitinib in head and neck is not supported by the results of this trial.”
	**Cohort A:** PS 0-1				**Cohort A:** n = 15				**Cohort A:** 8.3% (n = 1)		**Cohort A:** 25% (n = 3)			**Cohort A:** 21.1 weeks		
					**Evaluated patients:** n = 12											
	**Cohort B:** PS 2				**Cohort B:** n = 7				**Cohort B:** 0% (n = 0)		**Cohort B:** 29% (n = 2)			**Cohort B:** 19.1 weeks		
														1-year OS		
					**Evaluated patients:** n = 6									**Cohort A:** 22%		
														**Cohort B:** 14%		
41	Sunitinib monotherapy	II	2008, France and Belgium	Second	n = 38	Median 103 days	RECIST	N/A	N/A	N/A	47% (n = 18)	21% (n = 8)	N/A	Median	Median	“Sunitinib demonstrated modest activity in palliative SSCHN. The severity of some of the complications highlights the importance of improved patient selection for future studies with sunitinib in head and neck cancer. Sunitinib should not be used outside clinical trials in SSCHN.”
														102 days (95% CI 81-123)	60 days (95% CI 39-81)	
42	Sunitinib monotherapy	II	N/A, Greece	First	**Total:** n = 17	Median 12.8 months	RECIST	N/A	N/A	N/A	18% (n = 3)	65% (n = 11)	N/A	Median 4.0 months	N/A	“According to our findings, sunitinib monotherapy was not proven active in RM-SCCHN, and no further development of the drug in this indication is warranted.”
					**Evaluated patients:** n = 14						(95% CI 3.8–43.4%)	(95% CI 38.3–85.79%)		(95% CI 3.2–4.9)		
43	Semaxanib (SU5416) monotherapy	II	2002, USA	Second	**Total:** n = 35	N/A	WHO	N/A	N/A	N/A	19% (n = 6)	N/A	N/A	Median	N/A	“Treatment with SU5416 in patients with head and neck cancers is feasible, but objective responses are rare. Studies evaluating more potent anti-angiogenic agents in this disease are of interest.”
					**Evaluate d patients:** n = 31									6.25 months		
44	Semaxanib (SU5416) with paclitaxel	IB	2002, USA	Second	n = 12	N/A	RECIST	N/A	N/A	N/A	25% (n = 3)	58% (n = 7)	N/A	N/A	N/A	“Although the future development of SU5416 as a chemotherapeutic agent is unclear, there was a clinical benefit seen with this combination in 36% of the patients. This trial supports the use of developing antiangiogenic combinations, using molecular targeted agents, in head and neck carcinoma.”

CDR, disease control rate; CI, confidence interval; CR, Complete response rate; HR, hazard ratio; N/A, not available; NPC, nasopharyngeal carcinoma; ORR, overall response rate; OS, overall survival; PD, progressive disease; PFS, progression free survival; PR. Partial response rate; RECIST, Response Evaluation Criteria in Solid Tumours; SCCHN, squamous cell carcinoma of the head and neck; SD, stable disease; WHO, World Health Organization criteria.

Semaxinib was studied twice among a total of 47 patients, in one study as a monotherapy and in another in combination with paclitaxel, both as second-line treatment (**Table 3**). As a monotherapy, a dose of 145 mg/m^2^ was administered twice daily for 8 weeks, while in combination with paclitaxel at a dose of 110 mg/m^2^ on days 1, 15, 22 and 25, for a total of 42 cycles (43, 44). Semaxinib as a monotherapy had a median OS of 6.25 months with no severe toxicities. In combination with paclitaxel, SD was reported in 3/12 (25%) and PD in 7/12 (58%) patients (43, 44). **Supplementary Table 2** summarises the toxicity analyses of sunitinib and semaxanib.

### Other Tyrosine Kinase Anti-Angiogenesis Inhibitor Drugs

Lenvatinib, a multikinase inhibitor against VGFR1-3, was approved by US FDA and European Union to treat several solid cancers including thyroid cancer, renal cell carcinoma, and hepatocellular carcinoma (45). In addition to its role in inhibiting VEGFR1-3, it inhibits PDGFR-α, c-Kit, and the RET proto-oncogene (46). Lenvatinib was studied twice as a combination therapy with pembrolizumab among a total of 36 patients (**Table 4**). Both studies showed promising results and manageable safety profile. Chen et al., reported an ORR of 28.6% (95% CI 5.0-52.2) with median OS of 6.2 months (95% CI 2.9-9.6) (12). On the other hand, Taylor et al., reported a higher ORR which reached to 46% (95% CI 24.4-67.8) (13).

**Table 4:** Summary of the other tyrosine kinase anti-angiogenesis inhibitors clinical trials for head and neck squamous cell carcinoma.

Reference, clinical trial	Intervention	Phase	Completion year, Country	Treatment line	No. of patients	Follow-up	Evaluation criteria	CR	PR	ORR	SD	PD	DCR	OS	PFS	Conclusion
12	**Lenvatinib** in combination with pembrolizumab	N/A	NA, Taiwan	Second or more	n = 14	Median 2.8 months	RECIST 1.1	0.0% (n = 0)	28.6% (n = 4)	28.6% (n = 4)	14.3% (n = 2)	57.1% (n = 8)	42.9% (n = 6)	Median 6.2 months	Median 4.6 months	“Our study provided up-to-date evidence that pembrolizumab/lenvatinib combination therapy achieved objective responses in both heavily pretreated and anti-PD-1 refractory R/M HNSCC patients. This study supported the use of pembrolizumab/lenvatinib combination therapy in R/M HNSCC patients without standard of care.”
										(95% CI 5.0-52.2)			(95% CI 17.0-68.8)	(95% CI 2.9-9.6)	(95% CI 0.05-9.2)	
13	**Lenvatinib** in combination with pembrolizumab	Ib/II	2018, USA	First, second or more	n = 22	N/A	RECIST	5% (n = 1)	41% (n = 9)	46%	46% (n = 10)	0% (n = 0)	N/A	N/A	Median 4.7 months	“Lenvatinib plus pembrolizumab demonstrated a manageable safety profile and promising antitumor activity in patients with selected solid tumor types.”
NCT02501096										95% CI 24.4-67.8)					(95% CI 4.0-9.8)	
47		I	2009, USA	First	**Total:** n = 33	Median 19.8 months	RECIST 1.0		N/A		N/A	N/A		1-year OS	1-year OS	“Vandetanib with CRT was feasible.”
NCT00450138					**Evaluated pateints:** n = 30									96.9% (95% CI 91-100)	96.9% (95% CI 91-100)	
	**Regimen 1: Vandetanib** with radiotherapy				**Regimen 1:** n = 12			**Regimen 1:** 92%		**Regimen 1: Vandetanib 100mg:** 100.0%			**Regimen 1: Vandetanib 100mg:** 100.0%			
										**Vandetanib 200mg:** 100.0%			**Vandetanib 200mg:** 100.0%			
	**Regimen 2: Vandetanib** with radiotherapy and cisplatin				**Regimen 2:** n = 18			**Regimen 2**: 100%		**Regimen 2: Vandetanib 100mg:** 86.7%			**Regimen 2: Vandetanib 100mg:** 86.7%			
										**Vandetanib 200mg:** 66.7%			**Vandetanib 200mg:** 66.7%			
48	**Control group:** Docetaxel	II	2009, USA	Second	**Total:** n = 29	N/A	RECIST	**Control group:** 0%	**Control group:** 7% (n = 1)	**Control group:** 7% (n = 1)	**Control group:** 21% (n = 3)	**Control group:** 50% (n = 7)	**Control group:** 28.6% (n = 4)	Median weeks	Median weeks	“Although an initial benefit in response was noted and statistical criteria met there was only a minor trend towards improved PFS for the combined arm. The study was designed with low threshold for activity in each arm and results were deemed not to be of enough clinical significance in this group of patients to continue accrual.”
					**Control group:** n = 14									**Control group:** 26.8	**Control group:** 3.21	
										(95% CI 0.2-33.8%)				(95% CI 17.7-100.7)	(95% CI 3.0–22.0)	
	**Vandetanib group: Vandetanib** with docetaxel				**Vandetanib group:** n = 15			**Vandeta-nib group:** 0%	**Vandeta-nib group:** 13% (n = 2)	**Vandeta-nib group:** 13% (n = 2)	**Vandeta-nib group:** 47% (n = 7)	**Vandeta-nib group:** 33% (n = 5)	**Vandeta-nib group**: 60% (n = 9)	**Vandeta-nib group:** 24.1	**Vandeta-nib group:** 9	
										(95% CI 1.6-40.4%)				(95% CI 16.4-171.1)	(95% CI (5.86–18.1)	
49	**Axitinib** monotherapy	II	2015, China	Second or more	**Total:** n = 40	Median 28.3 months	RECIST 1.0	N/A	n = 1	N/A	n = 22	N/A	N/A	Median 10.4 months	Median 5.0 months	“Axitinib achieved durable disease control with a favorable safety profile in heavily pretreated NPC patients.”
					**Evaluated patients:** n = 37									(95% CI 6.8-19.0)	(95% CI 3.9-5.7)	
														1-year OS		
														46.3%		
50	**Axitinib** monotherapy	II	N/A, USA	Second or more	n = 30	N/A	RECIST 1.0	0%	7% (n = 2)	7% (n = 2)	70% (n = 21)	23.3% (n = 7)	76.7% (n = 23)	Median 10.9 months	Median 3.7 months	“Treatment with single agent axitinib should be considered due to acceptable toxicity profile and favorable median overall survival compared to standard therapies.”
														(95% CI 6.4-17.8)	(95% CI 3.5-5.7)	
51	**Pazopanib** daily with cetuximab weekly	I	2017, USA	Second	n = 31	Median 9.5 months	RECIST 1.1	6% (n = 2)	29% (n = 9)	35% (n = 11)	45% (n = 14)	19% (n = 6)	N/A	Median 9.5 months (95% CI 8.1-13.9)	N/A	“Pazopanib oral suspension at a dose of 800 mg/day was feasible to administer in combination with standard weekly cetuximab for patients with recurrent or metastatic HNSCC. Encouraging preliminary antitumour activity was observed with this combination therapy and warrants further validation in randomised trials.”
52	**Pazopanib** monotherapy	II	2009, Singa-pore	Second or more	n = 33	N/A	RECIST	0%	6.1% (n = 2)	N/A	48.5% (n = 16)	33.3% (n = 11)	N/A	Median 10.8 months	1-year PFS	“Pazopanib showed encouraging activity in heavily pretreated nasopharyngeal carcinoma with an acceptable toxicity profile.”
														(95% CI 8.6-21.8)	13%	
														1-year OS		
														44.4%		
53, NCT01462474	**Famitinib** with concurrent chemoradio-therapy (cisplatin)	I	2016, China	First	n = 20	Median 44 months	RECIST 1.1	CR after comple-tion of CCRT: 65.0% (n = 13)	PR in famitinib mono-therapy: 15% (n = 3)	N/A	SD in famitinib mono-therapy: 80% (n = 16)	PD in famitinib mono-therapy: 5% (n = 1)	N/A	N/A	1-year	“The recommended famitinib dose for phase II trial is 20 mg with CCRT for patients with local advanced NPC. Further investigation is required to confirm the effects of famitinib plus chemoradiotherapy.”
															PFS 85%	
															2-year	
															PFS 70%	
															3-year PFS	
															70%	
									PR after completion of CCRT: 35.0% (n = 7)							
54	**Foretinib** montherapy	II	2009, USA	Second	n = 14	Patients were contact-ed for follow-up at 90 and 180 days after the last dose.	RECIST 1.0	0%	0%	0%	n = 7	n = 3	n = 7	Median 5.59 months	Median 3,65 months	“The efficacy results, prolonged disease stabilization and tolerable side-effect profile, support further investigation, possibly in combination with other targeted agents or cytotoxic chemotherapy for SCCHN.”
										(95% CI 0-23.2)	50%	24.1%	50%			
													(95% CI 23.0–77.0)	(95% CI 3.71 - NA)	(95% CI 3.4-5.3)	
55	**m-510** with gemcitabine and cisplatin	I	2003, Nether-lands	Third or more	n = 13	N/A	N/A	N/A	n = 3	N/A	n = 8	N/A	N/A	N/A	N/A	“Combining ABT-510 at doses of 50 mg and 100 mg with gemcitabine–cisplatin is feasible.”

CCRT, concurrent chemoradiotherapy; CDR, disease control rate; CI, confidence interval; CR, complete response rate; CRT, chemoradiation; HNSCC, head and neck squamous cell carcinoma; HR, hazard ratio; N/A, not available; NPC, nasopharyngeal carcinoma; ORR, overall response rate; OS, overall survival; PD, progressive disease; PD-1, programmed cell death protein; 1 PFS, progression free survival; PR. partial response rate; RECIST, Response Evaluation Criteria in Solid Tumours; SD, stable disease; WHO, World Health Organization criteria.

Vandetanib, a multikinase inhibitor, was approved by the US FDA to treat symptomatic or progressive, unresectable or metastatic medullary thyroid cancer (56). It binds to VEGF and epidermal growth factor (EGF) receptor families as well as RET (rearranged-during-transfection), BRK (breast tumour kinase), TIE2 (receptor-like tyrosine kinase) and Src (proto-oncogene tyrosine-protein kinase) receptors (56). Vandetanib was studied twice as a combination therapy among a total of 62 patients (**Table 4**), but the conclusions from these studies were inconclusive. As a first-line treatment, vandetanib in combination with radiotherapy as a first-line treatment resulted in an ORR of 100.0% (95% CI 61.0–100.0%) (47). When combined with radiotherapy and cisplatin, vandetanib yielded ORRs of 86.7% (100-mg vandetanib) and 66.7% (200-mg vandetanib; 95% CI 62.1–96.3% and 30.9–90.3%, respectively) (47). As a second-line treatment, the combination of vandetanib and docetaxel exhibited insufficient clinical significance (48). The safety profile of vandetanib in both studies was feasible.

Axitinib is a multitarget TKI approved by the US FDA for the treatment of renal cell carcinoma (57). Axitinib inhibits VEGFR-1, - 2 and -3, PDGFR-α, PDGFR-β and c-KIT (58). Axitinib was studied twice among a total of 70 patients as second-line treatment for metastatic or recurrent HNSCC (**Table 4**). In both studies, monotherapy with a continuous dose of 5–10-mg twice daily was administered. The median OS reached 10.4 and 10.9 months, respectively, and treatment was well-tolerated (49, 50).

Pazopanib is a small-molecule multikinase inhibitor that inhibits VEGF receptors (1, 2 and 3), PD-ECGFR-α and -β and the stem-cell factor receptor (SCF) c-KIT (59). Several countries have approved pazopanib for the treatment of advanced soft-tissue sarcoma and renal cell carcinoma (60). Two trials on pazopanib were included here, carried out among a total of 64 patients and both using pazopanib as the second- or more-line treatment of metastatic or recurrent HNSCC (**Table 4**). The trials used a daily pazopanib dose between 200–800 mg. One phase II trial studied pazopanib as a monotherapy and reported a safety profile with a PR of 6.1% and 1-year OS and PFS of 44.4% and 13%, respectively (52). In a phase I trial, pazopanib was combined with cetuximab, achieving CR in 6% and PR in 29% of patients. ORR was 35% and the safety profile was acceptable (51).

Famitinib is a receptor TKI that binds to several RTKs including VEGFR-2 and -3, SCF receptor c-KIT and PDGFR (61). One phase I trial among a total of 20 patients studied famitinib as a monotherapy and in combination with cisplatin and radiotherapy as a first-line treatment (famitinib at an initial dose of 12.5 mg/day, increased to 16.5, 20.0 and 25.0 mg/day) (53). Famitinib as a single agent was well-tolerated, with a PR for famitinib monotherapy of 15% and, after completion of treatment with chemoradiotherapy, increasing to 35%. CR was achieved in 65% of patients after completing treatment. PFS at 1-, 2- and 3-years follow-up reached 85%, 70% and 70%, respectively (53).

Foretinib, an experimental drug not yet in clinical use, is a small molecule that inhibits hepatocyte growth factor (HGF) receptor c-MET and VEGFR-2 (62). Foretinib was studied as a single agent in one phase II trial among 14 patients, at a dose of 240 mg for 5 days on a 14-day treatment cycle. ORR was 0% (95% CI 0–23.2%) with a median OS and PFS of 5.59 and 3.65 months, respectively (95% CI 3.71–NA and 3.4–5.3 months). The side effects were tolerable (54).

ABT-510 is a synthetic peptide that inhibits several pro-angiogenic growth factors including VEGF, bFGF, HGF and interleukin 8 (IL-8) (63). One trial among 13 patients examined ABT-510 in combination with gemcitabine–cisplatin chemotherapy in later lines of metastatic HNSCC (50–100-mg dose of ABT-510 twice daily) (55). Treatment was well- tolerated and PR was reported in 3/13 (23%) and SD in 8/13 (62%) patients (55).

**Supplementary Table 2** summarises the toxicity analyses of the drugs discussed above.

### Endostatin

Endostatin is a broad-spectrum angiogenesis inhibitor approved by the State Food and Drug Administration of China for the treatment of non-small cell lung cancer (64), but has not enjoyed approval by other regulatory authorities. Endostatin is a naturally occurring protein, featuring a 20-kDa fragment of type XVIII collagen (65). Endostatin inhibits endothelial cell proliferation, migration/invasion and tube formation, and appears to bind to a variety of receptors, including VEGFR-2 and -3, integrin α_5_β_1_ and α_V_β_3_ and Glypican-1 and -4 (65, 66).

Endostatin was studied in 3 clinical trials among 186 patients (**Table 5**), in two trials in combination with chemotherapy (cisplatin, paclitaxel, 5-fluorouracil or gemcitabine) and in one trial with radiotherapy (67–69). In one study, recombinant human endostatin adenovirus (E10A) was administered at a dose of 1.0 × 10^12^ virus particles on days 1 and 8 for 4 cycles (69). In the other trials, a dose of 15 mg/day was administered for 14 days (67, 68). When endostatin was added to radiotherapy as a first-line treatment, CR was achieved in 60.0% and PR in 40.0% of patients compared with a CR of 61.5% and PR of 38.5% in the control group (radiotherapy with cisplatin) (67). Survival rates improved with endostatin: 2-year OS and PFS rates reached 100% in the endostatin group compared to 69.6% and 67.3% in the control group (radiotherapy with cisplatin) (67). Endostatin in combination with cisplatin and gemcitabine as a second-line treatment yielded an ORR of 85.7% and 1-year OS and PFS rates of 90.2% and 69.8%, respectively (68). In the study with E10A, the effect of endostatin in combination with cisplatin and paclitaxel proved beneficial in patients with HNSCC compared to the control group (chemotherapy only) (69). ORR with E10A was 39.7% compared to 29.9% in the control group (p=0.154; chemotherapy only) (69). The median OS was 19.10 months in the E10A group and 14.53 months when chemotherapy alone was administered [p=0.366, HR 0.79 (95% CI 0.47–)] (69). Across all trials, endostatin was well-tolerated with no significant systemic toxicity, revealing promising anticancer effects when administered as a combination therapy. **Supplementary Table 2** summarises the toxicity analysis of endostatin.

**Table 5:** Summary of the endostatin clinical trials for head and neck squamous cell carcinoma.

Reference, clinical trial number	Intervention	Phase	Completion year, Country	Treatment line	No. of patients	Follow-up	Evaluation criteria	CR	PR	ORR	SD	PD	DCR	OS	PFS	Conclusion
67		N/A	2016, China	First	**Total:** n = 23	Median years	RECIST			N/A	N/A	N/A	N/A	2-year OS	2-year PFS	“The present study demonstrates that, compared with IMRT combined with chemotherapy, IMRT combined with endostar has similar efficacy in the treatment of locally advanced NPC, but significantly weaker acute adverse reactions, which improve the life quality of NPC patients.”
	**Control group:** Radiotherapy with concurrent cisplatin				**Control group:** n = 13	**Control group:** 49		**Control group:** 61.5% (n = 8)	**Control group:** 38.5% (n = 5)					**Control group:** 69.6%	**Control group:** 67.3%	
						**E10A group:** 59										
	**Endostar group:** Radiotherapy with endostar				**Endostar group:** n = 10			**Endostar group:** 60.0% (n = 6) p	**Endostar group:** 40.0% (n = 4)					**Endostar group:** 100.0%	**Endostar group:** 100.0%	
68	Endostar with gemcitabine and cisplatin	II	2014, China	Second	n = 28	Median 56 years	RECIST	50.0% (n = 14)	35.7% (n = 10)	85.7% (n = 24)	3.6% (n = 1)	10.7% (n = 3)	89.3%	1-year OS 90.2%	1-year PFS 69.8%	“Our results of this study suggest that a combination of Endostar with GC chemotherapy can lead to effective tumour regression, control disease progression, and improve prognosis in M NPC. Therefore, a combined Endostar and GC regimen should be considered as a potential treatment for patients with M NPC.”
NCT01612286															Median PFS 19.4 months	
										(95% CI 66.42-95.31)			(95% CI 70.65-97.20)		(95% CI 13.6–25.1 months)	
69		II	2010, China	Not stated	**Total:** n = 135	Median years	RECIST							Median months	Median months	“In summary, E10A plus chemotherapy is a safe and effective therapeutic approach in patients with advanced head and neck squamous cell carcinoma or nasopharyngeal carcinoma.”
NCT00634595	**Control group:** Cisplatin and paclitaxel				**Control group:** n = 67	**Control group:** 51		**Control group:** 1.5% (n = 1)	**Control group:** 28.4% (n = 19)	**Control group:** 29.9% (n = 20)	**Control group:** 52.2% (n = 35)	**Control group:** 19.4% (n = 13)	**Control group:** 80.6% (n = 54)	**Control group:** 14.53 months	**Control group:** 3.60 months	
	**E10A group:** Cisplatin and paclitaxel with endostatin (E10A)				**E10A group:** n = 68	**E10A group:** 53		**EA10 group:** 4.4% (n = 3)	**EA10 group:** 35.3% (n = 24)	**E10A group:** 39.7% (n = 27)	**E10A group:** 55.9% (n = 38)	**E10A group:** 7.4% (n = 5)	**E10A group:** 92.6% (n = 63)	**E10A group:** 19.10 months	**E10A group:** 7.03 months	

CDR, disease control rate; CI, confidence interval; CR, Complete response rate; HR, hazard ratio; IMRT, intensity-modulated radiation therapy; N/A, not available; NPC, nasopharyngeal carcinoma; ORR, overall response rate; OS, overall survival; PD, progressive disease; PFS, progression free survival; PR. Partial response rate; RECIST, Response Evaluation Criteria in Solid Tumours; SD, stable disease; WHO, World Health Organization criteria.

## Discussion

Angiogenesis plays a crucial role in tumour growth, invasion and metastasis, while the overexpression of VEGF in HNSCC associates with advanced disease and a poor prognosis (70, 71). Several therapeutic agents have been developed to target angiogenesis pathways, although they have yet to receive approval for the treatment of HNSCC. In this systematic review, we summarised the published data regarding bevacizumab, TKIs and endostatin in HNSCC clinical trials.

Bevacizumab has been approved by the US FDA to treat several malignancies as a monotherapy or in combination with chemo- or radiotherapies (15). Preclinical data point towards encouraging results with bevacizumab in HNSCC as well, since an *in vitro* study on HNSCC cell lines showed that bevacizumab decreased VEGF secretion (72). In another study on the xenografts of HNSCC cell lines, bevacizumab was tested in combination with radiation, resulting in significant decreases in angiogenesis, the inhibition of tumour growth and an increase in tumour cell apoptosis compared to radiation alone (73). In HNSCC clinical trials, bevacizumab was the most frequently studied drug and was analysed in several combinations as well. In some trials, significant toxicities were reported (16, 17, 26), although in other studies, the same combinations appeared well-tolerated with encouraging results (11, 18, 19, 21–25, 27). Three categories of combinations were used in the trials: (1) bevacizumab in combination with erlotinib and chemotherapy/chemoradiotherapy (16, 23, 27); (2) bevacizumab in combination with cetuximab and chemotherapy/chemoradiotherapy (17, 18, 21) and (3) bevacizumab in combination with chemotherapy or chemoradiotherapy (11, 19, 20, 22, 24, 26). Significant toxicities, such as a perforation, fistula, diarrhoea, mucositis, dysphagia, haemorrhage and hematologic toxicity, were reported in one trial from all of these treatment combinations, and no further trials were recommended (16, 17, 26). Other studies described more promising results and encouraging ORR or survival rates. Bevacizumab was also the only drug that had progressed to a phase III trial. For instance, in 2019, results from a large phase III trial were published (11), and the addition of bevacizumab significantly improved both PFS and ORR, although a statistically significant improvement to OS was not achieved. Unfortunately, the addition of bevacizumab associated with a higher rate of treatment-related grade 3–5 bleeding events (6.7% vs. 0.5%; p<0.001) and treatment-related deaths (9.3% vs. 3.5%; p=0.022) (11).

Famitinib, a TKI, tended to be the most promising experimental drug. It was studied in one trial as an initial monotherapy for two weeks, immediately followed by its use in combination with cisplatin and radiotherapy (53) among patients with stage III–IV HNSCC. Famitinib was well- tolerated and, in combination with chemoradiotherapy, CR was achieved in 65% of patients and 1-, 2- and 3-year PFS reached 85%, 70% and 70%, respectively (53). However, the lack of comparison group limits the generalisability of these results. Some TKIs yielded inconsistent results in various trials and the findings remain inconclusive. Vandetanib showed varying results with an ORR of 13% (PR in 2/15 patients) with docetaxel following progression to platinum-based therapy (48). In a curative setting, combining vandetanib with radiotherapy yielded 100% ORR, while when combined with radiotherapy and cisplatin, it yielded an ORR of 86.7% (at a dose of 100-mg vandetanib) and 66.7% (at a dose of 200-mg vandetanib), respectively (47). Sorafenib and sunitinib were both well-tolerated, although the therapeutic effects of either drug remained modest (31–35, 39–42).

Preclinical studies with endostatin demonstrated the suppression of HNSCC cell migration and invasion, as well as high levels of cell apoptosis and reduced tumour angiogenesis (74–76). Based on our systematic review, endostatin emerged as the most promising drug for inhibiting angiogenesis in HNSCC clinical trials with feasible safety profiles and promising anticancer effects. Endostatin was analysed in three Chinese trials, with encouraging ORR and survival rates reported. The combination of endostatin with cisplatin and gemcitabine yielded an ORR of 85.7% (68). When endostatin was added to radiotherapy, similar response and survival rates were achieved in a small first-line study compared to chemoradiotherapy, although significantly fewer acute adverse events were reported in the endostatin arm (67). Furthermore, endostatin received approval for the treatment of NSCLC in China, but not for HNSCC (65). One phase II trial of endostatin was completed in the US on patients with advanced neuroendocrine tumours, although no significant tumour regression was reported (77).

Immunotherapy is the newest treatment modality for HNSCC patients. Based on boosting the patient’s own immune system to eliminate cancer cells, immunotherapy surpasses conventional chemotherapy in its specificity and decreases therapy-related morbidities. Two immunotherapies, pembrolizumab and nivolumab, have received FDA and European Medicines Agency (EMA) approval to treat HNSCC (78, 79). Only one angiogenesis inhibitor, lenvatinib, was tested in combination with immunotherapy, pembrolizumab, and gave a promising anti-tumour activity in two trials (12, 13). Interestingly, two out of ten patients who had failed previous anti-PD-1 therapy, achieved partial response when they received pembrolizumab/lenvatinib combination therapy.

Across all 38 trials included in this systematic review, only one was in phase III, while the others were in phase I (8), II (24), I/II (2) or unspecified (2). Bevacizumab was included in the largest number of trials, and the phase III trial was also the largest study consisting of a total of 403 patients (11). The other trials enrolled only 10 to 70 participants. Trials with TKIs featured small study populations, comprising only 10–40 patients each. In addition, one large phase II study on endostatin consisted of a total of 153 patients (69). The studies examined featured a variety of different comparison groups and a variety of previous treatment lines and, thus, direct interstudy comparisons should be avoided. Further extensive randomised trials, particularly with well-tolerated endostatin and a combination of lenvatinib with immunotherapy, are truly needed in order to gain a clearer understanding of their benefit in HNSCC patients.

## Conclusions

Angiogenesis is a hallmark of tumour progression and targeting angiogenesis has proved successful as a cancer treatment approach in some solid tumours. Although the clinical benefit of angiogenesis inhibitors in treating HNSCC patients remains unclear and they associate with considerable toxicity, few trials gave encouraging results. Further clinical studies are still needed to evaluate which, if any, angiogenesis inhibitors are beneficial to patients with advanced HNSCC. Specifically, further data are needed to identify the most effective combinations with other adjuvant therapies such as immunotherapy, especially with regards to identifying those patients who will benefit most from treatment.

## Data Availability Statement

The original contributions presented in the study are included in the article/**Supplementary Material**. Further inquiries can be directed to the corresponding author.

## Author Contributions

AH designed the study, performed the literature search, extracted the data and wrote the manuscript. WW performed the literature search and extracted the data. OV performed the literature search and extracted the data. KS designed the study and interpreted the results. PK designed the study and interpreted the results. TS designed the study and interpreted the results. AA-S designed the study, interpreted the results and supervised the work. All authors contributed to the article and approved the submitted version.

## Funding

The authors gratefully acknowledge the following funders of this study: the Sigrid Jusélius Foundation, the Cancer Society of Finland, the Oulu University Hospital MRC grant, Helsinki University Central Hospital research funds, the Jane and Aatos Erkko Foundation, and the Medicinska Understödsföreningen Liv och Hälsa Foundation.

## Conflict of Interest

The authors declare that the research was conducted in the absence of any commercial or financial relationships that could be construed as a potential conflict of interest.

## Abbreviations

BRK, Breast tumour kinase; CDR, Disease control rate; CI, Confidence interval; CR, Complete response rate; CSF1R, Colony stimulating factor receptor type 1; EGF, Epidermal growth factor; EMA, European Medicines Agency; FLT3, Foetal liver tyrosine kinase receptor 3; GDNF, Glial cell line–derived neutrophilic factor receptor; HGF, Hepatocyte growth factor; HNSCC, Head and neck squamous cell carcinoma; HPV, Human papillomavirus; HR, Hazard ratio; NSCLC, Non-squamous non-small cell lung cancer; ORR, Overall response rate; OS, Overall survival; PD, Progressive disease; PD-1, Programmed cell death protein; PDGFR, Platelet-derived growth factor receptor; PFS, Progression-free survival; PR, Partial response rate; RCC, Renal cell carcinoma; RECIST, Response Evaluation Criteria in Solid Tumours; RET, Rearranged-during-transfection; SD, Stable disease; Src, Proto-oncogene tyrosine-protein kinase; TIE2, Receptor-like tyrosine kinase; TKIs, Receptor tyrosine kinase inhibitors; US FDA, US Food and Drug Administration; VEGF, Vascular endothelial growth factor; WHO, World Health Organisation criteria.
