# Supplementary material for: Angiogenesis Inhibitors for Head and Neck Squamous Cell Carcinoma Treatment: Is There Still Hope?
Source: Front Oncol. 2021 Jun 14;11:683570. doi: 10.3389/fonc.2021.683570 (PMC8236814; doi:10.3389/fonc.2021.683570)
Supplement: Supplementary file 1 [file Table_1.docx]

**Supplementary Table 1. Inclusion and exclusion criteria.**

| INCLUSION CRITERIA | EXCLUSION CRITERIA |
| --- | --- |
| - Patients with head and neck squamous cell carcinoma - Completed clinical trials - Angiogenesis inhibitors as part of the treatment - As outcome response rates and/or survival rates and toxicity | - Other conditions than head and neck squamous cell carcinoma - In vitro studies, animal models or other studies that are not clinical trials - Language other than English |
