# Supplementary material for: Angiogenesis Inhibitors for Head and Neck Squamous Cell Carcinoma Treatment: Is There Still Hope?
Source: Front Oncol. 2021 Jun 14;11:683570. doi: 10.3389/fonc.2021.683570 (PMC8236814; doi:10.3389/fonc.2021.683570)
Supplement: Supplementary file 2 [file Table_2.docx]

**Supplementary Table 2. Summary of the toxicity of the angiogenesis inhibitors.**

| **DRUG,**  **REFERENCE** | **CONCLUSION ABOUT TOXICITY** |
| --- | --- |
| **ABT-510**  Gietema et al. 2006. | “Pharmacokinetic interactions were not observed and adding ABT-510 does not appear to increase toxicity.” |
| **Axitinib**  Hui et al. 2018. | “Grade 3/4 toxicities were uncommon, including hypertension (8%), diarrhea (5%), weight loss (5%), and pain (5%). All hemorrhagic events were grade 1 (15%) or grade 2 (3%). Elevated diastolic blood pressure during the first 3 months of axitinib treatment was significantly associated with improved overall survival (HR, 0.29; 95% CI, 0.13–0.64, P = 0.0012). Patient-reported fatigue symptom was associated with hypothyroidism (P = 0.039). Axitinib PK parameters (Cmax and AUC(0-t)) were significantly correlated with tumor response, toxicity, and serum thyroid-stimulating hormone changes.” |
| **Axitinib**  Swiecicki et al. 2015. | “Among all 42 enrolled patients, axitinib was reasonably well tolerated”. |
| **Bevacizumab**  Ahn et al. 2018. | “A total of 46% of all patients were unable to complete protocol treatment. GI toxicities including perforation, fistula, and diarrhea have presented themselves as severe and unexpected complications. Although MTD in this study was defined at an erlotinib dose of 50 mg, the high rates of toxicity in all dose levels lead us to recommend against advancement of this regimen to a phase II trial.” |
| **Bevacizumab**  Argiris et al. 2013. | “Grade 3–4 adverse events were expected and occurred in less than 10% of patients.” |
| **Bevacizumab**  Argiriss et al. 2019  NCT00588770 | “There was increased toxicity, including a higher rate of treatment-related grade 3 to 5 bleeding events (6.7% v 0.5%; P < .001) and treatment-related deaths (9.3% v 3.5%; P = .022) with BC versus chemotherapy.” |
| **Bevacizumab**  Argiris et al. 2016  NCT0070397 | “Serious toxicities associated with cetuximab–RT, including mucositis and dysphagia, were comparable between arms. Hemorrhage was significantly more common on the bevacizumab arm. Other class toxicities associated with anti-angiogenic therapy, including gastrointestinal perforation and wound-healing complication, were rare but only occurred in the bevacizumab arm.” |
| **Bevacizumab**  Cohen et al. 2009.  NCT00055913 | “The most common toxicities of any grade were rash and diarrhea (41 and16 of 48 subjects, respectively). Three patients experienced serious bleeding events.” |
| **Bevacizumab**  Fury et al. 2016. | “This phase II study describes safety and encouraging efficacy with the addition of two monoclonal antibodies, bevacizumab and cetuximab, to two cycles of cisplatin administered concurrently with intensity-modulated radiation therapy in locally/regionally advanced HNSCC. Both planned cycles of bevacizumab were completed by 93% of study subjects, and all completed the planned radiation therapy. The most common ≥ grade 3 adverse events were lymphopenia, functional mucositis, and dysphagia. “ |
| **Bevacizumab**  Fury et al. 2012. | “Adverse events associated with the study regimen were similar to those described in prior studies of concurrent radiation and high‐dose cisplatin for this patient population, although myelosuppression appeared to be increased compared with prior studies of cisplatin monotherapy.” |
| **Bevacizumab**  Hainsworth et al. 2011. | “As expected, grade 3/4 mucosal toxicity occurred frequently (88%) during combined modality; no unexpected toxicity resulted from the addition of bevacizumab and erlotinib.” |
| **Bevacizumab**  Lee et al. 2012 | “No Grade 3 or 4 hemorrhages were observed in this trial. Nine patients had grade 1 and 1 grade 2 hemorrhage. Besides hemorrhage, no other unusual toxicities associated with the addition of bevacizumab to cisplatin and/or fluorouracil was noted in this group of patients. In terms of worst non-hematologic toxicity, 5 patients (11.4%) experienced grade 4 while the majority of the patients (79.5%) experienced grade 3 toxicity.” |
| **Bevacizumab**  Nyflot et al. 2015. | “All patients experienced grade 3 toxicity, but no dose-limiting toxicities or significant bleeding episodes were observed.” |
| **Bevacizumab**  Salama et al. 2011. | “The addition of bevacizumab to FHX numerically increased acute toxicity compared with patients treated with FHX alone. In our prior phase I study, no ≥grade 3 hematologic toxicity was seen in patients treated with the BFH doses used in this study. Although mucositis and dermatitis rates were not increased, all patients experiencing ≥grade 3 acute hematologic toxicity were treated with BFHX. Additionally, all on treatment deaths during or shortly after treatment occurred in the BFHX arm. Small patient numbers limit statistical comparisons. Furthermore, patients undergoing surgical procedures were prone to need many operations for nonhealing wounds and graft revisions.” |
| **Bevacizumab**  Yao et al. 2015  NCT00281840 | “The most common local toxicities were mucositis and dermatitis. Two patients developed hemorrhage. There was no grade 5 toxicity.” |
| **Bevacizumab**  Yoo et al. 2012.  NCT00140556 | “The current study shows acceptable safety and encouraging efficacy with the integration of dual EGFR and VEGF inhibitors with CRT in locally advanced nonmetastatic HNC. The increased incidence of osteoradionecrosis and soft tissue necrosis may be associated with the use of bevacizumab.” |
| **Endostatin**  Jin et al. 2013.  NCT01612286 | “The hematologic events were those most experienced by the patients in this study, including two patients with febrile neutropenia. The most common nonhematologic events were hyponatremia, fatigue, nausea, and vomiting. One patient had grade II left ventricular diastolic dysfunction.” |
| **Endostatin (Endostar)**  Kang et al.  2018. | “IMRT combined with endostar resulted in significantly lower grades of leucopenia,nausea/vomiting, weight loss, and oral mucositis compared with IMRT combined with chemotherapy. The grades of late adverse reactions of IMRT combined with endostar were not different from those of IMRT combined with chemotherapy.” |
| **Endostatin (E10A)**  Ye et al. 2014.  NCT00634595 | “Except for fever, no adverse events were associated with the E10A treatment.” |
| **Famitinib**  Chen et al. 2018.  NCT01462474 | “Neither radiotherapy interruptions nor deaths occurred during the study. Famitinib as a single agent was generally well tolerated. More adverse events were observed with famitinib plus CCRT.” |
| **Foretinib**  Seiwert et al. 2013. | “Foretinib 240 mg on a 5/9 schedule wasgenerally well tolerated.” |
| **Lenvatinib**  Chen et al. 2021. | “In total, adverse events of all grades were noted in 77% of patients, and grade 3 or 4 adverse events were noted in 29% of patients. The most frequent adverse events of any grade included anemia (79%), hypertension (57%), and elevated alanine transaminase (36%). Severe adverse events included hypertension in two cases (14%), infection in one case (7%), and thrombocytopenia in one case (7%). No treatment-related deaths were noted in our cohort.” |
| **Lenvatinib**  Taylor et al. 2020. | “The safety profile of lenvatinib plus pembrolizumab was consistent with that observed in prior lenvatinib and pembrolizumab monotherapy trials, with no unexpected adverse events. In general, toxicities were manageable with supportive care medications, treatment interruption and discontinuation, and/or lenvatinib dose reductions.” |
| **Pazopanib**  Adkins et al. 2018. | “Adverse events attributable to pazopanib were different to those attributable to cetuximab. In this trial, the non-overlapping adverse events of the combination were well tolerated. Dose-limiting toxic events attributed to pazopanib included neutropenia, proteinuria, and fatigue. The most common grade 3 adverse events attributable to pazopanib across all cycles were hypertension, anaemia, fatigue, hypoalbuminaemia, neutropenia, and diarrhoea. Grade 4 adverse events or treatment-related deaths did not occur.” |
| **Pazopanib**  Lim et al. 2011. | “Common grade 3/4 toxicities included fatigue (15.2%), hand-foot syndrome (15.2%), anorexia (9.1%), diarrhea (6.1%), and vomiting (6.1%).” |
| **Semaxanib**  **(SU5416)**  Cooney et al. 2005. | “The combination of SU5416 with paclitaxel had a higher than expected incidence of thromboembolic events and prophylactic anticoagulation should be considered for future trials that combine an angiogenesis inhibitor with cytotoxic chemotherapy.” |
| **Semaxanib**  Fury et al. 2007. | “The most common toxicity was headache, and 20 of 35 patients required dose reductions.”  “The risk of hemorrhage for HNSCC patients receiving antiangiogenic therapy with SU5416 appears to be low. One patient experienced a fatal carotid artery bleed which likely was due to local progression of disease, although we cannot rule out drug-related toxicity.” |
| **Sorafenib**  Elser et al. 2007. | ”Overall, sorafenib was well tolerated in this patient population. On the basis of the percentage of cycles, the most commonly encountered toxicities of all grades were fatigue (79%), lymphopenia (42%), mucositis/stomatitis (42%), anemia (35%), hand-foot skin reaction (29%), and hypertension (28%). No grade 4 toxicities were observed.” |
| **Sorafenib**  Gilbert et al. 2015. | “Overall, the regimen was well tolerated.” |
| **Sorafenib**  Lalami et al. 2016 NCT00199160 | “Regarding the safety profile, all toxicities that had been reported are usual during therapy with sorafenib. However, severe fatigue, hyponatremia, and hypophosphatemia were more frequent in this study population  when compared with previous studies with sorafenib.” |
| **Sorafenib**  Williamson et al. 2010. | “Sorafenib was well tolerated.” |
| **Sunitinib**  Choong et al. 2010. | “Sunitinib was well tolerated in this study but efficacy parameters in cohort A were not met at interim analysis and the study was closed prior to completing planned accrual.” |
| **Sunitinib**  Hui et al. 2011. | “Hemorrhagic events (all grades) occurred in nine patients (64%), all from bleeding sources in the upper aerodigestive tract, including epistaxis in six patients, hemoptyses in three patients and hematemesis in two patients. Two patients died of hemorrhagic events. The high incidence of hemorrhage from the upper aerodigestive tract in NPC patients who received prior high-dose RT to the region is of concern.” |
| **Sorafenib**  Xue et al. 2013. | “In conclusion, our study results revealed that the combination of sorafenib, cisplatin (80 mg/m2), and 5-FU (3000 mg/m2) was a tolerable and feasible regimen in recurrent or metastatic NPC” |
| **Sunitinib**  Fountzilas et al. 2010. | “Overall, treatment was well tolerated.”  “Grade 3 side effects were infrequently observed, apart from fatigue, probably due to the short duration of the sunitinib treatment.” |
| **Sunitinib**  Machiels et al.  2010. | “The most frequent grade 1 to 2 toxicities were as follows: anemia (58%), fatigue (42%), hypertension (42%), mucositis (37%), anorexia (34%), thrombocytopenia (29%), diarrhea (26%), skin rash (21%), and tumor bleeding (18%). The main grade 3 to 4 toxicities were fatigue (32%), anorexia (16%), thrombocytopenia (13%), diarrhea (8%), and left ventricular ejection fraction decrease (5%). Additionally, six patients, five of whom had locoregional relapse, experienced grade 3 to 5 arterial head and neck bleeds (one grade 3, one grade 4, and four grade 5). All of these patients, excluding the one with the grade 4 bleed, had received prior radiation to the head and neck area. The grade 3 to 5 bleeds occurred spontaneously without instrumentation or concurrent significant thrombocytopenia.” |
| **Vandetanib**  Limaye et al. 2013. | “Most common adverse events were fatigue, dysphagia, diarrhea or constipation, cytopenias and alopecia.” |
| **Vandetanib**  Papadimitrakopoulou et al. 2016.  NCT00450138 | “In conclusion, vandetanib appeared to be generally well tolerated at a dose of 100 mg/day when administered with radiotherapy and cisplatin.” |

**AUC:** Area Under the Curve, **BC:** Bevacizumab, **BFH:** Bevacizumab (B) with 5-Fluorouracil (5-FU), and Hydroxyurea (HU), **BFHX:** Bevacizumab (B) with 5-Fluorouracil (5-FU), Hydroxyurea (HU), and Radiotherapy, **CCRT:** Concurrent Chemo-Radiotherapy, **CI:** Confidence Interval , **Cmax :** Maximum Serum Concentration , **CRT:** Chemo-Radiotherapy, **EGFR:** Epidermal Growth Factor Receptor, **FHX:** 5-FU, Hydroxyurea And Concomitant Radiation For 5 Days, **GI:** Gastrointestinal, **HNC:** Head and Neck Cancer, **HR:** Hazzard Ratio, **IMRT:** Intensity-Modulated Radiation Therapy, **MTD:** Maximum Tolerated Dose, **NPC:** Nasopharyngeal Carcinoma, **PK:** Pharmacokinetics, **RT:** Radiotherapy, **VEGF:** Vascular Endothelial Growth Factor, **5-FU:**  5-Fluorouracil.
